# Non-Coding RNAs: Uncharted Mediators of Thyroid Cancer Pathogenesis

**DOI:** 10.3390/cancers12113264

**Published:** 2020-11-04

**Authors:** Hossein Tabatabaeian, Samantha Peiling Yang, Yvonne Tay

**Affiliations:** 1 Cancer Science Institute of Singapore, National University of Singapore, Singapore 117599, Singapore; csiht@nus.edu.sg; 2 Endocrinology Division, Department of Medicine, National University Hospital, Singapore 119228, Singapore; 3 Department of Medicine, Yong Loo Lin School of Medicine, National University of Singapore, Singapore 117597, Singapore; 4 Department of Biochemistry, Yong Loo Lin School of Medicine, National University of Singapore, Singapore 117597, Singapore

**Keywords:** thyroid carcinoma, non-coding RNA, radioactive iodine, drug resistance, prognosis

## Abstract

**Simple Summary:**

Thyroid cancer is the most common type of endocrine system malignancy. The effective diagnosis, precise treatment, and better short and long-term prognosis of thyroid cancer patients have remained challenging. Non-coding RNAs (ncRNAs) are emerging molecules with diverse capabilities in initiating and promoting thyroid cancer upon dysregulation. The expression profile of these molecules could be used to detect thyroid cancer, determine the therapeutic approaches, and predict the patients’ survival. Thus, ncRNAs could have clinical significance in precision medicine.

**Abstract:**

Thyroid cancer is the most prevalent malignancy of the endocrine system and the ninth most common cancer globally. Despite the advances in the management of thyroid cancer, there are critical issues with the diagnosis and treatment of thyroid cancer that result in the poor overall survival of undifferentiated and metastatic thyroid cancer patients. Recent studies have revealed the role of different non-coding RNAs (ncRNAs), such as microRNAs (miRNAs), long non-coding RNAs (lncRNAs) and circular RNAs (circRNAs) that are dysregulated during thyroid cancer development or the acquisition of resistance to therapeutics, and may play key roles in treatment failure and poor prognosis of the thyroid cancer patients. Here, we systematically review the emerging roles and molecular mechanisms of ncRNAs that regulate thyroid tumorigenesis and drug response. We then propose the potential clinical implications of ncRNAs as novel diagnostic and prognostic biomarkers for thyroid cancer.

## 1. Introduction

Thyroid carcinoma is the most prevalent malignancy of the endocrine system with a significantly higher incidence in women [1,2,3] and is the 9th most common cancer globally [3]. The incidence of thyroid cancer has been escalating worldwide in recent years [2,4]. According to the Surveillance, Epidemiology and End Results (SEER) Program [5], ~53,000 newly diagnosed cases of thyroid cancer are anticipated in the United States in 2020 and these will represent 2.9% of all new cancer cases. Globally, there were newly diagnosed 567,233 thyroid cancer cases in 2018 that comprise 3.1% of all cancer incidences [3]. The cause of this rise in incidence is multi-factorial. It is partially contributed by the detection of incidental thyroid cancers with the increasing use of cross-sectional imaging. However, other factors likely play a role in the pathogenesis of these thyroid cancer cases. The well-described risk factors of thyroid cancer are exposure to ionizing radiation, particularly in childhood, and family history of thyroid cancer. The potential role of other factors such as smoking, obesity, hormonal exposures, and environmental toxins/ endocrine-disrupting chemicals have been implicated [6]. Their mechanistic role in the pathogenesis of thyroid cancer has not been well-elucidated. Further environmental and biomolecular studies will be essential to better understand the complex etiology of thyroid cancer [7].

The thyroid gland comprises two specific cell types. Thyroid follicular (epithelial) cells produce and secrete the thyroid hormones, thereby regulating the body’s temperature, metabolism, and heart rate. The second cell type is parafollicular cells (C cells) that reside in the thyroid connective tissue and are responsible for calcitonin secretion to regulate the levels of calcium and phosphate in the body [8,9]. Thyroid cancer is classified into four types based on histopathological analysis and site of origin: (1) papillary thyroid carcinoma (PTC) that accounts for 80% of all cases and commonly metastasize to cervical lymph nodes. (2) Follicular thyroid carcinoma (FTC) represents 10% of all cases and the widely invasive sub-type has a tendency for metastasis to distant sites, whereas its minimally invasive sub-type has an overall low risk of recurrence and metastasis. (3) Medullary thyroid carcinoma (MTC) that is more aggressive than PTC and FTC, has higher metastatic rates to cervical lymph nodes and distant sites, and occurs in 4% of thyroid cancer cases. (4) Poorly differentiated thyroid carcinoma (PDTC) and anaplastic thyroid carcinoma (ATC) are the most aggressive and least differentiated types of thyroid carcinoma with the highest rate of spread to other organs. PDTC and ATC account for 5–10% of follicular thyroid cancers [1,10,11]. Among these types, PTC, FTC, PDTC and ATC originate from follicular cells, whereas MTC develops from C cells.

The main measure to detect thyroid cancer is ultrasound, which can be complemented with radioiodine scanning and fine-needle aspiration (FNA) biopsy [12]; and the treatment for thyroid cancer is surgical excision [13]. Post-surgery, other therapeutic strategies including radioactive iodine (RAI) therapy, and systemic therapies might be incorporated into the treatment regime for patients with high risk of recurrent or persistent disease [14]. Improvements in detection and treatment strategies have improved the survival of thyroid cancer. According to the American Society of Clinical Oncology, the 5-year survival for most of the non-metastatic thyroid cancer types is above 95%, whereas this rate falls drastically to 31% for ATC. In metastatic thyroid cancer, however, the 5-year survival rate for PTC, FTC and MTC drops to 78, 63 and 39%, respectively. The 5-year survival rate for metastatic ATC cases is only 4%. These data indicate that there is room for improvement in the current measures for thyroid cancer management, especially for more aggressive subtypes, and highlight the need to identify specific molecular diagnostic/prognostic biomarkers for personalized medicine.

**Figure 1:**
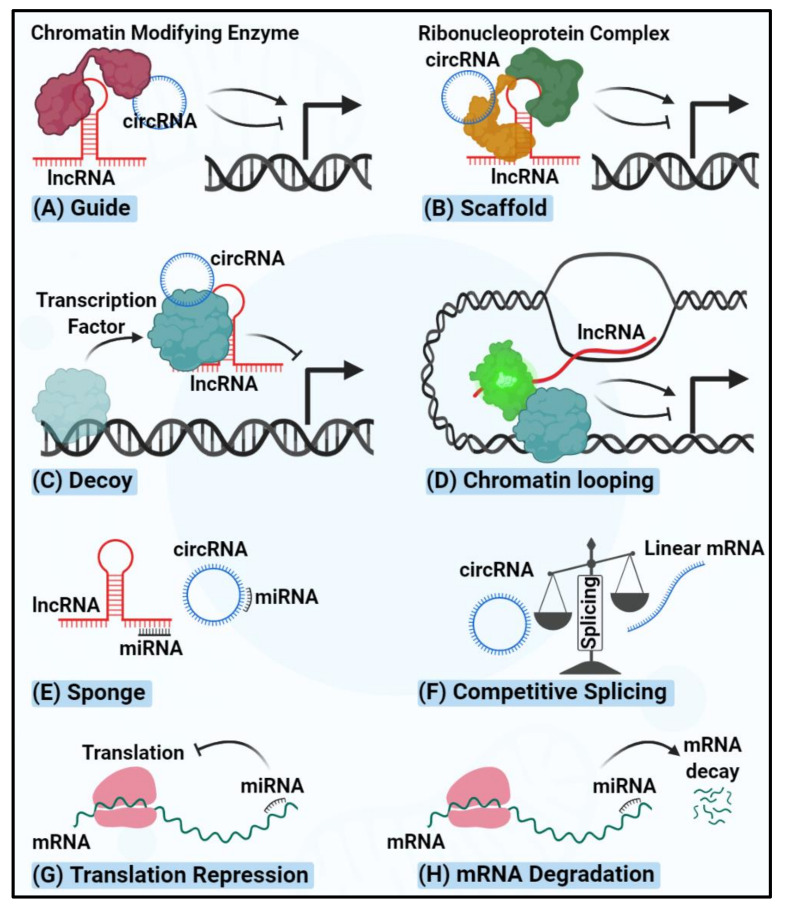
The schematic of mechanisms of action of ncRNAs. (**A**) lncRNAs and circRNAs can guide chromatin remodeling factors to either activate or repress the transcription of target genes. (**B**) lncRNAs and circRNAs, as scaffolds, can facilitate the assembly of ribonucleoprotein complexes to either activate or repress the transcription of target genes. (**C**) lncRNAs and circRNAs can sponge the transcription factors to repress the transcription of the target genes. (**D**) Upon transcription, lncRNAs can facilitate the formation of regulatory complexes and loop the DNA, thereby priming long-range gene transcription. (**E**) lncRNAs and circRNAs can sponge the miRNAs, thereby rescuing the miRNA target transcripts. (**F**) circRNAs can compete with the linear mRNA(s) transcribed from their host gene and repress the canonical splicing over the back splicing. (**G**) miRNAs bind to their target mRNAs and repress the translation efficiency upon non-perfect complementation between the seed region and targeted binding site. (**H**) miRNAs bind to their target mRNAs and result in transcript degradation upon perfect complementation between the seed region and targeted binding site.

Current diagnostic biomarkers in thyroid cancer include point mutations in the BRAF, NRAS, KRAS, HRAS genes, and rearrangements in paired box 8 (PAX8)/peroxisome proliferator-activated receptor gamma (PPARG) and RET [15,16,17,18]. In addition, mutations within the telomerase reverse transcriptase (TERT) promoter region have been detected more frequently in aggressive thyroid cancer cases [19]. Notwithstanding, the present molecular testing of thyroid cancer is chiefly useful to stratify indeterminate nodules, thereby to avoid surgery or prevent unnecessary repeated FNA [20]. The identification of novel molecular biomarkers to facilitate early diagnosis and predict drug responsiveness would be invaluable to improve the survival rate and quality of life for thyroid cancer patients. Non-coding RNAs (ncRNAs) are fast emerging as novel functional molecules and also biomarkers in human cancers [21,22,23,24]. Around 98% of the transcriptome in human cells corresponds to ncRNAs that are transcribed from previously considered “junk DNA” sequences such as introns and intergenic nucleotides [25]. These non-protein-coding transcripts are classified as small ncRNAs (sncRNAs) and long non-coding RNAs (lncRNAs) that are less than or more than 200 nucleotides in length, respectively. sncRNAs comprise small nuclear RNAs (snRNAs), microRNAs (miRNAs), piwi interacting RNAs (piRNAs) and small nucleolar RNAs (snoRNAs) [26,27,28,29,30]. However, circular RNAs (circRNAs) belong to both sncRNA and lncRNA classifications due to their variable length, ranging from 100–10,000 nucleotides [31]. The majority of human miRNAs and circRNAs are transcribed from introns, reflecting the importance of non-coding DNA sequences in determining cell fate [32]. Compelling evidence highlights the involvement of ncRNAs in almost all physiological and biological cell processes, such as cell growth, proliferation, senescence, apoptosis, invasion, migration, angiogenesis and inflammation [22,33,34,35,36,37,38]. ncRNAs impose their functions through different mechanisms—summarized in the following sections and Figure 1.

Upon dysregulation, oncogenic and tumor suppressor ncRNAs could drive cancer initiation and progression, and/or alter drug response [39,40,41]. This review systematically focuses on the recent advances in the molecular roles of different ncRNA classes in thyroid cancer, as well as their implications as diagnostic and prognostic biomarkers and therapeutic targets.

## 2. miRNAs Regulating Thyroid Carcinogenesis

miRNAs are a subclass of sncRNAs with about 19–24 nucleotides in length that mainly regulate gene expression at the post-transcriptional level. miRNAs bind to miRNA response elements on target protein-coding and non-coding transcripts to regulate their expression. miRNA binding may lead to mRNA degradation or translational repression, depending on the extent of complementarity with each target region [42,43,44] (Figure 1). miRNAs have also been shown to regulate genes transcriptionally via interacting with lncRNAs, leading to the up or down-regulation of target genes [45,46,47]. The impact of miRNA dysregulation on cancer was first described by Calin et al. in 2002 [48]. Shortly after, He et al. showed the up-regulation of miR-221, miR-222, and miR-146 in PTC patients, which are the first dysregulated miRNAs reported in thyroid cancer [49]. To date, many additional studies have identified miRNAs involved in thyroid cancer initiation and progression [50,51]. Here, we summarize the recent list of dysregulated miRNAs in thyroid cancer, published from 2015 onward, with implications in cancer initiation and progression, briefly listed in Table 1.

**Figure 2:**
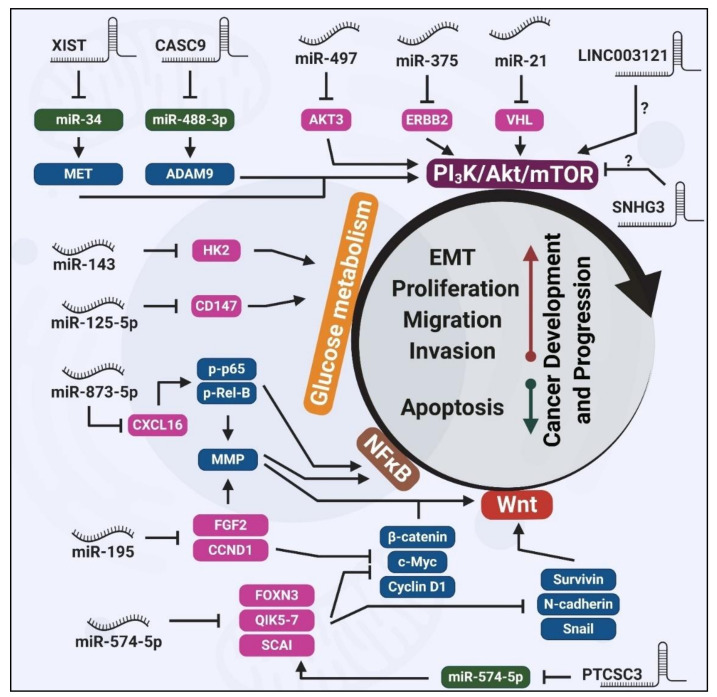
Schematic overview of ncRNA involvement in thyroid cancer-related signaling pathways. Key components of the PI_3_K/Akt/mTOR pathway, such as AKT3, ERBB2 and VHL, are regulated by different miRNAs and lncRNAs in thyroid cancer. The metabolism of glucose is regulated by miR-143 and miR-125-5p. Well-known cancer-related pathways, NFκB and Wnt, are tightly regulated by miRNAs and lncRNAs in thyroid cancer. Dysregulation of these miRNAs and lncRNAs in various types of thyroid cancer eventually results in the induction of proliferation, migration and invasion, while apoptosis is suppressed. Note: Pink represents direct targets of miRNAs, green represents direct miRNA targets of lncRNAs and blue represents the indirect target of miRNA or lncRNAs.

A group of dysregulated miRNAs has been shown by in vitro and in vivo assays to inhibit apoptosis and promote proliferation, invasion and migration in thyroid cancer. For instance, the down-regulation of miR-524-5p [68], miR-141-3p [61], miR-9 [67], miR-199b-5p [66], miR-1266 [60], miR-144 [62], miR-150 [64] and miR-7 [69] were demonstrated to play pivotal roles in thyroid tumorigenesis. However, there is lack of inclusive data to know how these alterations could mechanistically impose tumorigenic properties in the context of thyroid cancer. Despite this ambiguity, the dysregulation of some other miRNAs has been reported to promote thyroid carcinomas via different signaling pathways, such as Wnt and phosphatidylinositol-4,5-Bisphosphate 3-Kinase (PI_3_K)/Akt (Figure 2).

### 2.1. Wnt-Mediated Tumorigenic Effects of Dysregulated miRNAs

Wnt signaling pathway is essential to embryonic development and tissue homeostasis. Hyperactivation of Wnt signaling is abundant in human cancer [71,72] and thyroid carcinoma is not an exception [73]. miR-574-5p is one of the most well-studied miRNAs that is linked to thyroid carcinomas, FTC and PTC in particular, via Wnt pathway. Up-regulation of miR-574-5p was reported by different groups to promote proliferation, Epithelial-Mesenchymal Transition (EMT), invasion, migration and inhibit apoptosis [55,74,75]. miR-574-5p exerts its oncogenic effects via targeting Forkhead Box N3 (FOXN3), Quaking protein 5–7 (QKI5–7), and Suppressor of Cancer Cell Invasion (SCAI) transcripts. Collectively, this results in the activation of the Wnt signaling pathway. This, in turn, up-regulates β-catenin, N-cadherin (a mesenchymal biomarker), Snail, c-Myc, cyclin D1 and survivin proteins [55,74,75], leading to downstream oncogenic phenotypes and thyroid cancer development.

In addition, the down-regulation of miR-195, which is shown in different cancers [76,77,78,79,80], was reported to cause thyroid tumorigenesis in vitro and in vivo via activating Wnt pathway. Through direct targeting cyclin D1 and fibroblast growth factor 2 (FGF2) genes, miR-195 was shown to inhibit cell proliferation, migration, and invasion via down-regulating β-catenin, c-Myc, cyclin D1 and matrix metallopeptidase-13 (MMP-13) [65]. A number of Wnt signaling pathway inhibitors are being examined in clinical trials, such as frizzled receptor antagonist, Vantictumab [81]. It is conceivable that Wnt pathway blockade may neutralize the oncogenic effects of miR-574-5p or miR-195 up-regulation in thyroid cancer, leading to the suppression of cancer development or progression. This hypothesis is yet to be tested experimentally.

### 2.2. PI_3_K/Akt-Mediated Tumorigenic Effects of Dysregulated miRNAs

PI_3_K/Akt signaling is a well-established oncogenic signaling pathway in multiple human cancers [82], including thyroid carcinoma [83]. Thus far, dysregulation of several miRNAs has been reported to regulate thyroid cancer by targeting the PI_3_K/Akt signaling pathway. For instance, miR-21 overexpression was shown to target Von Hippel-Lindau (VHL) tumor suppressor and activate PI_3_K/Akt pathway. This, in turn, resulted in an increase in the EMT markers N-cadherin and vimentin, and promote cell proliferation and invasion [54]. PTEN is another validated target of miR-21, reported in various human cancers, that plays pivotal role in the inhibition of PI_3_K/Akt pathway [84,85,86]. Thus, miR-21 up-regulation could synergistically suppress PI_3_K/Akt pathway via targeting VHL and PTEN transcripts. This needs to be examined experimentally. In addition to miR-21, heightened expression of miR-625-3p was reported to activate PI_3_K/Akt pathway via an unknown mechanism [56]. miR-497 [67] and miR-375 [57] were demonstrated to be down-regulated in thyroid cancer and target AKT3 and ErbB2 receptor tyrosine kinase 2 (ERBB2), respectively. The latter is reported to be overexpressed in PTC clinical samples [87] and mediates the resistance to mitogen-activated protein kinase (MAPK) inhibitors in BRAF-mutant thyroid cancer cell lines [88], implying that ERBB2 could drastically affect the thyroid tumorigenesis. The AKT3 up-regulation, caused by miR-497 down-regulation, resulted in the activation of PI_3_K/Akt pathway that eventually promotes proliferation and invasion in thyroid cancer [67]. Although miR-375 down-regulation similarly led to augmentation of oncogenic properties in vitro and in vivo [57], it is unclear whether these effects were mediated by ERBB2, a key oncogenic protein involved in the PI_3_K pathway [89]. Besides, miR-96 is another example of overexpressed ncRNA in thyroid cancer with possible involvement in PI_3_K/Akt pathway. miR-96 imposes its proliferative and anti-apoptotic effects via targeting Forkhead Box O1 (FOXO1), a known tumor suppressor in thyroid cancer [90] that is phosphorylated and subsequently degraded by PI_3_K/Akt activation [91]. In parallel with PI_3_K/Akt, miR-96 could synergistically amplify the FOXO1-mediated oncogenic properties in thyroid cancer. However, this proposed model has not been experimentally tested.

Despite the advances in harnessing the oncogenic properties of thyroid cancer via dual administration of PI3K/Akt pathway inhibitors—palbociclib and omipalisib [92]—it is not known how dysregulated miRNAs in this context could determine the drug response.

### 2.3. Glucose Metabolism-Mediated Tumorigenic Effects of Dysregulated miRNAs

The higher activity of glycolytic pathway has been shown in thyroid cancer to impose tumorigenesis [93,94]. Of note, miR-143 and miR-125a-5p have been reported to affect thyroid tumorigenesis via regulating glucose metabolism [59,95]. Studies in vitro and in vivo demonstrated that miR-143 directly targets Hexokinase 2 (HK2) and thereby down-regulates its expression. miR-143-mediated HK2 down-regulation resulted in suppressed glycolysis and therefore, decreased proliferation and migration [95]. These support the tumor-suppressive role of miR-143; however, the expression profile of this miRNA remained to be investigated in clinical thyroid cancer samples. A more comprehensive study revealed that miR-125a-5p was down-regulated in both thyroid cancer cell lines and clinical samples. miR-125a-5p was shown to block glucose metabolism via direct targeting CD147 that eventually suppressed cell viability and migration [59]. Given the clinical significance of targeting glucose metabolism in cancer therapy [96,97], a more in-depth study to examine both miR-143 and miR-125a-5p may open a new avenue to control thyroid cancer via regulating glucose metabolism.

### 2.4. Dysregulated miRNAs in Other Signaling Pathways

The down-regulation of miR-873-5p was shown to increase the expression of its direct target, C-X-C Motif Chemokine Ligand 16 (CXCL16) in PTC cell lines. This resulted in an increase in phosphorylation of p65 and Rel-B, thereby leading to activation of the NFκB pathway., and consequently, up-regulation of MMP1, MMP9 and MMP13 proteins. The overall phenotypic effects of miR-873-5p down-regulation are enhanced thyroid cancer cell proliferation, migration and invasion [70], which is theoretically consistent with the lowered expression of this miRNA in PTC clinical samples.

Overexpression of miR-155 was shown by Zhang et al. in ATC aggressive cells to promote cell proliferation, invasion and migration via directly targeting suppressor of cytokine signaling 1 (SOCS1) transcripts [53]. SOCS1 is a ubiquitin ligase with the tumor-suppressive activity that is silenced in human tumors [98,99,100]. This protein was demonstrated to be a direct inhibitor of the catalytic activity of Janus kinase 1 (JAK1), JAK2 and tyrosine kinase 2 (TYK2), thereby suppressing the JAK/signal transducer and activator of transcription (STAT) signaling pathway [101]. Given the oncogenic nature of JAK/STAT pathway in different human cancers [102] including thyroid carcinomas [103], targeting miR-155 could inhibit the thyroid cancer development/progression via activating SOCS1 and suppressing JAK/STAT. Notwithstanding, this hypothesis has not been tested in thyroid cancer. The reduced expression of miR-30 and miR-200 in ATC tumors is another remarkable finding that can help distinguish the ATC from PTCs or FTCs. The down-regulation of miR-30 and miR-200 mediates the suppression of mesenchymal-epithelial transition (MET), while inducing EMT [104].

Although the dysregulation of dozens of miRNAs has been reported in thyroid cancer, the majority of these studies focus on specific miRNAs and do not include transcriptome-wide screening approaches. Hence, RNA-seq- and/or microarray-based methods could be utilized to show a clearer map of dysregulated miRNAs. Moreover, despite the comprehensive studies with beneficial attempts to unravel the underlying molecular pathways involved in thyroid cancer, more studies are needed to fully map the mechanisms by which miRNA dysregulation mediates thyroid cancer development and progression. These could reveal the master regulator miRNAs that control key thyroid cancer signaling pathways, which may be used in personalized medicine.

## 3. lncRNAs Regulating Thyroid Carcinogenesis

LncRNAs are a sub-class of ncRNAs >200 nucleotides in length. LncRNAs regulate target gene expression through different mechanisms. At the transcriptional level, lncRNAs can interact with the polycomb repressive complex 2 (PRC2) and confine its access to particular genomic regions, leading to the suppression of gene expression [67]. For example, HOX transcript antisense RNA (HOTAIR) suppresses homeobox D (HOXD) expression via interacting with the catalytic subunit of PRC2, called enhancer of zeste homolog 2 (EZH2) [105,106]. Alternatively, lncRNAs have been reported to recruit DNA methyltransferases to modify chromatin conformation [107,108,109]. As an example, PTEN pseudogene (PTENpg1) utilizes DNA methyltransferase 3A (DNMT3a) to regulate the transcription of PTEN [110]. LncRNAs can also regulate gene expression at the post-transcriptional level by complementary sequence-specific mechanisms that affect the mRNA splicing, turnover and translation [44]. For example, metastasis- associated lung adenocarcinoma transcript 1 (MALAT1) was shown to compete with splicing regulatory proteins for binding on target mRNAs [111]; BETA-SECRETASE 1-ANTISENSE (Bace1-AS) hybridizes with Bace1 mRNA to increase its half-life [112], and long intergenic non-coding RNA p21 (lincRNA-p21) recruits translation repressors to catenin beta 1 (CTNNB1) gene to silence it [113]. The schematic of lncRNAs regulatory mechanisms is shown in Figure 1.

A series of lncRNAs have been identified to be abnormally regulated and expressed in thyroid cancer (Table 2).

LncRNAs play roles in regulating diverse cellular processes [132], thereby causing cancer upon dysregulation [133].

The dysregulation of lncRNA H19 has been studied widely in different cancers such as bladder [134], breast [135,136] and liver [137]. The dysregulation of H19 in thyroid cancer is subject of controversy since the down-regulation was reported in FTC [126] and PTC [127], while the overexpression is reported in PTC, ATC [117] and PTC/PTC stem cells (PTCSCs) [118]. Given the functional studies performed in these reports, the oncogenic nature of H19 in thyroid cancer is more conceivable since H19 knockdown resulted in the suppression of proliferation, migration, and invasion in ATC cells in vitro and inhibited tumorigenesis and metastasis in vivo [117]. Moreover, the depletion of H19 was shown to suppress the sphere formation ability [118], collectively suggest that H19 acts as an oncogene in thyroid cancer.

In contrast to the many studies focusing on specific lncRNAs, Pellecchia et al. performed a transcriptome-wide screening using lncRNA microarray method. Comparing the ATC tumor to the normal samples revealed that Prader Willi/Angelman Region RNA5 (PAR5) was a significant down-regulated lncRNA in tumors. Mechanistically, overexpression of PAR5 expression resulted in the down-regulation and dissociation of EZH2, leading to the relieving of E-cadherin transcription efficiency, which subsequently reduced proliferation and migration of ATC-derived cells [129]. In another unbiased study, utilizing the chromatin immunoprecipitation (ChIP)-seq method, Linc00941 was found as a highly expressed enhancer-associated lncRNA in PTC tumor samples as compared to the paired healthy tissues. Of note, Linc00941 expression was significantly higher in BRAFV600E PTC patients and correlated with extrathyroidal extension in PTC patients, suggesting that the up-regulation might be mediated by BRAFV600E. However, this hypothesis was not tested. Functionally, Linc00941 promoted the proliferation and invasion of PTC cell lines, hypothetically mediated by targeting Cadherin 6 CDH6 transcripts [122].

The up-regulation and corresponding oncogenic properties of a diverse range of lncRNAs are reported in different studies, including distal-less homeobox 6-antisense 1 (DLX6-AS1) [115], nuclear paraspeckle assembly transcript 1 (NEAT1) [67], ENST00000539653.1 (ENS-653) [116], Unc-5 netrin receptor B-antisense 1 (UNC5B-AS1) [124], LINC00514 [121], LINC00152 [120], MALAT1 [123], HLA complex P5 (HCP5) [119] and n340790 [119]. Although the exact modes of action by which these lncRNAs promote/suppress thyroid carcinomas have not been elucidated, we summarize a number of altered lncRNAs with implications in Wnt and PI_3_K/Akt signaling pathways (Figure 2).

### 3.1. Wnt-Mediated Tumorigenic Effects of Dysregulated lncRNAs

The putative role of lncRNAs in thyroid tumorigenesis mediated by activating the Wnt signaling pathway has been also tested. Thyroid carcinoma susceptibility candidate 3 (PTCSC3) down-regulation was observed in the clinical samples as well the cell lines resulting in elevation of miR-574-5p and subsequently down-regulation of SCAI and activation of β-catenin (Figure 2). Functionally, overexpression of PTCSC3 inhibited the cell proliferation and migration via suppressing the Wnt pathway. Besides, PTCSC3 suppressed the growth in vivo, indicating that PTCSC3 acts as a tumor suppressor in thyroid cancer [55]. It is unclear whether inhibiting/overexpressing the dysregulated lncRNAs could affect the downstream targetome of thyroid cancer-related signaling pathways. For example, could PTCSC3 overexpression suppress the Wnt activity using TopFlash assay?

### 3.2. PI_3_K/Akt-Mediated Tumorigenic Effects of Dysregulated lncRNAs

In the context of the PI_3_K/Akt pathway, the down-regulation of small nucleolar RNA host gene 3 (SNHG3) was reported to promote growth and invasiveness in vitro and in vivo via activating PI_3_K/Akt/mechanistic target of rapamycin kinase (mTOR) pathway [131]. However, the mechanism by which such phenotype was observed has not been elucidated. Cancer susceptibility 9 (CASC9) is another example of a dysregulated ncRNA in thyroid cancer. The up-regulation of this lncRNA was reported in PTC patient tissues and cell lines and mechanistically was shown to sponge miR-488-3p to rescue ADAM metallopeptidase domain 9 (ADAM9) oncogene. This consequently resulted in promoting the proliferative, migrative, and invasive abilities of thyroid cancer cells in vitro, and augmenting the tumorigenesis in vivo via activating epidermal growth factor receptor (EGFR)/PI_3_K/Akt pathway [114].

Similarly, the up-regulation of X-inactive specific transcript (XIST) in thyroid cancer was demonstrated to activate the PI_3_K/Akt pathway via sponging miR-34a and the subsequent rescue of MET, a well-known oncogene in thyroid cancer. The functional experiments properly proved the oncogenic role of XIST, where its silencing suppressed the proliferation and tumor growth in vitro and in vivo [125].

LINC003121 is an example of down-regulated lncRNA in thyroid cancer. Although the mechanistic evaluations were not performed, the lower expression of LINC003121 was shown to increase PI_3_K and p-Akt expression, leading to increased cell proliferation and invasion in vitro, and promoting tumorigenicity in thyroid cancer xenograft models in nude mice [128]. Together with reviewed miRNAs, PI_3_K/Akt pathway plays a pivotal role in thyroid development and progression upstream of a myriad of ncRNAs (Figure 2).

Taken together, recent evidence highlights the molecular and functional relevance of different lncRNAs in thyroid cancer, especially in the context of PI_3_K/Akt and Wnt signaling pathways. However, it remained to know how and to what extend the alterations in lncRNAs could influence the Wnt and/or PI_3_K-mediated therapy. Besides, high throughput methods, such as RNA-seq or ChIP-seq and microarray, have not been utilized to uncover the dysregulated lncRNAs in thyroid cancer patients in a transcriptome-wide manner.

## 4. circRNAs Regulating Thyroid Carcinogenesis

circRNAs, the stably expressed ncRNAs in different cell types with special annular structures, play fundamental regulatory roles in the physiological processes of the cell and have implications in human diseases such as cancer. Mechanistically, circRNAs impose their downstream effects via sponging miRNAs or interacting with proteins [138,139,140] (Figure 1). A few of oncogenic circRNAs have been reported to drive thyroid cancer (Table 3).

For instance, the up-regulation of circular forkhead box protein M1 (circFOXM1) was reported in PTC tissues, as well as ATC and PTC cell lines [143]. With no effect on the linear FOXM1 transcript, circFOXM1 was demonstrated to modulate cancer progression through sponging miR-1179 and rescuing high mobility group box protein 1 (HMGB1) expression, which eventually promotes tumor growth of PTC in vitro and in vivo [143]. Likewise, the up-regulation of circ_0008274 has been reported in PTC tissues and cell lines. High circ_0008274 expression has been associated with more advanced thyroid cancer TNM staging and lymph node metastases. The in vitro studies revealed that this circRNA promoted cell proliferation and invasion. circ_0008274 imposes its effect via the activation of the mammalian target of rapamycin (mTOR) signaling pathway (increasing p-mTOR) and the inhibition of the 5′ AMP-activated protein kinase (AMPK) (reducing p-AMPK) [141]. Given the known negative regulatory effect of AMPK on mTOR protein [144], it is conceivable that circ_0008274 activates mTOR pathway via inhibiting AMPK protein, leading to thyroid cancer development and progression. However, this notion remained to be tested in depth in thyroid cancer cell lines.

Collectively, circRNAs have been shown to be deregulated and promote thyroid cancer mainly through sponging miRNAs. More studies are needed, particularly in ATC and metastatic thyroid cancers, to develop deeper insights on circRNAs roles in thyroid cancer initiation and promotion.

## 5. ncRNAs May Regulate the Biology of Thyroid Tumor Microenvironment

The tumor microenvironment is composed of a variety of tumor-associated immune cells as well as growth factors, cytokines, and chemokines. Changes in the tumor microenvironment that occurs during cancer progression could induce various biological processes like angiogenesis, proliferation, invasion and metastasis, immune tolerance and alter the response to therapeutic agents [145,146]. B cells, T cells, mast cells, dendritic cells and macrophages are the main immune cells accumulated in the tumor microenvironment, where machrophages are characterized by plasticity and diversity and play an important role in the immune response [147]. In response to hypoxia, tumor-associated macrophages produce WNT7b, which in turn attributes to up-regulation of vascular endothelial growth factor (VEGF) by adjacent vascular endothelial cells in the tumor microenvironment. This eventually results in the elevated angiogenesis and promotion of tumor growth [148,149]. Of note, the secretion of diverse chemokines and cytokines recruits the regulatory T lymphocytes that result in the inhibition of effector T cells [150]. Although there is lack of conclusive evidence about the roles of ncRNAs in regulating the microenvironment-associated cells/components in thyroid cancer, here we review the current data and propose the potential ncRNA-related mechanisms.

NEAT1, an up-regulated lncRNA in thyroid cancer, has been reported to induce tumor-associated macrophages via sponging miR-214 and inducing the β-catenin/Wnt signaling pathway [151]. This further highlights the therapeutic potential of NEAT1 as a potential target for thyroid cancer therapy [152]. Another highly-expressed lncRNA in thyroid cancer, MALAT1, can activate angiogenesis via increasing fibroblast growth factor 2 (FGF2) expression in tumor-associated macrophages [153]. Another up-regulated lncRNA, PTCSC3, may also regulate the thyroid cancer tumor microenvironment via activating the Wnt signaling pathway [55].The up-regulation of miR-574-5p and miR-195 has been discussed in Section 2.1 to induce proliferation, EMT, invasion and migration via activating the Wnt pathway in thyroid cancer. miR-574-5p and/or miR-195 overexpression could induce the chemokines/cytokines and activate the tumor-associated immune cells. This hypothesis remained to be tested to assess the potential of these miRNAs in modulating the thyroid tumor microenvironment.

## 6. ncRNAs are Novel Candidates for Early Detection of Thyroid Cancer

In poorly differentiated, medullary, and anaplastic thyroid carcinomas [154], early detection of cancer is key to maximizing the chance of successful treatment and prolonging patient survival. This is achieved by systematic screening and recognizing the warning signs [155]. For thyroid cancer, the current standard of care is to perform neck ultrasonography together with FNA cytology, which are determinants to discriminate benign and malignant thyroid nodules [156]. However, 10–40% of thyroid nodule FNA cytology results are indeterminate, resulting in a repeat FNA several months later [157,158]. In indeterminate thyroid FNA cytology, the estimated incidence of malignancy ranges between 10 and 75% [159]. Furthermore, it is not possible to distinguish the benign follicular adenoma (FA) from the malignant FTC via FNA cytology unlike other forms of thyroid cancers. Hence, the diagnosis of malignancy in this group can only be made post-surgery via histology that demonstrates the presence of capsular or vascular invasion. This difficulty in making a definite diagnosis for cytologically-indeterminate FNA usually results in delayed definitive treatment and management. Treatment for repeatedly indeterminate cases usually takes the form of a diagnostic hemi-thyroidectomy, or a two-stage thyroidectomy for patients with thyroid cancer on diagnostic histology. Furthermore, the majority of patients (76–81%) with indeterminate cytology have benign thyroid nodules and thus were subjected to unnecessary diagnostic thyroid surgeries [160]. As such, there is a critical need for clinicians to improve the ability to predict the risk of thyroid cancer in thyroid nodules.

As reviewed in the introduction section, molecular tests for oncogene mutations such as BRAF, NRAS, KRAS, HRAS, Pax8-PPARG, re-arrangement of RET and TERT have been used to improve benign/malignant differentiation [6,161]. However, they have not increased the specificity of such diagnostic methods [162,163]. Moreover, the performance of these kits may also differ in different populations due to the different prevalence of the genetic alterations of interests. For example, while BRAF^V600E^ is present in 38–58% of thyroid cancers in patients of European ancestry, the prevalence across East Asia can range from 80% in Korea, 62% in China, and 53% in India [164,165,166,167,168,169]. These suggest that more molecular tools are needed, especially for the timely diagnosis of advanced thyroid cancers. Here, we focus on the significance of diagnostic ncRNAs in thyroid cancer (Table 4).

ncRNAs have tremendous potential as diagnostic biomarkers as they are found to be stable and detectable in body fluids [177,178,179]. For instance, miR-10b-5p, miR-195-5p, miR-132-3p, miR-20a3p, miR-185-5p, and miR-296-5p are reported to be significantly overexpressed in gastric cancer patients’ sera [180]. Commercially, a lncRNA named prostate cancer-associated 3 (PCA3) has been approved by the US Food and Drug Administration as a urine biomarker for prostate cancer [181].

The diagnostic significance of miR-222, miR-146a-5p and miR-146b has been shown by different studies. Zhang et al. studied the serum level of miR-222-3p, miR-17-5p, miR-451a, miR-146a-5p, miR-132-3p and miR-183-3p in PTC, MTC, benign nodule and control groups. The results revealed that the serum levels of miR-222-3p, miR-17-5p, and miR-451a were markedly increased, while miR-146a-5p, miR-132-3p, and miR-183-3p were significantly decreased in the PTC and benign nodule groups compared with the control group. There was no difference in the miRNA expression profile between the PTC and the benign nodule group. Nevertheless, the serum levels of miR-222-3p and miR-17-5p were significantly increased in the MTC group than the benign nodule and control groups. Therefore, they concluded that miR-222-3p and miR-17-5p can accurately discriminate MTC from the benign nodule group and healthy controls [172]. Rosignolo et al. studied the serum level of miR-146a-5p, miR-221-3p and miR-222-3p before and 30 days after surgery in PTC patients. The pre-surgical high expression of these miRNAs with a significant post-surgical down-regulation supported the diagnostic significance of miR-146a-5p, miR-221-3p and miR-222-3p [173]. Moreover, the heightened serum level of miR-222, miR-221, miR-146b and miR-21 was detected in the PTC tumors with <10 mm diameter as compared to benign nodules [174]. The importance of miR-146b was supported by another study that showed the escalated expression of this miRNA in FNA biopsy samples of PTC samples [171]. Graham et al. measured the expression level of miRNAs in the serum of PTC samples versus the benign nodule group. The results revealed that miR-146a-5p and miR-199b-3p were highly expressed in the PTC group, whereas let7b-5p and miR-10a-5p were down-regulated [175]. These various studies on the clinical significance of miRNAs have resulted in the advent of a commercial diagnostic product in thyroid cancer. ThyraMIR^®^ miRNA classifier product is developed to be used in combination with the conventional molecular diagnostic platform as a new thyroid cancer diagnostic tool, with promising outcomes showing 89% sensitivity and 85% specificity [170]. This kit is expected to reduce 85% of unnecessary surgeries of benign thyroid nodules [161,182,183,184]. The miRNAs utilized in this classifier are miR-29b-1-5p, miR-31-5p, miR-138-1-3p, miR-139-5p, miR-146b-5p, miR-155, miR-204-5p, miR-222-3p, miR-375 and miR-551b-3p.

**Figure 3:**
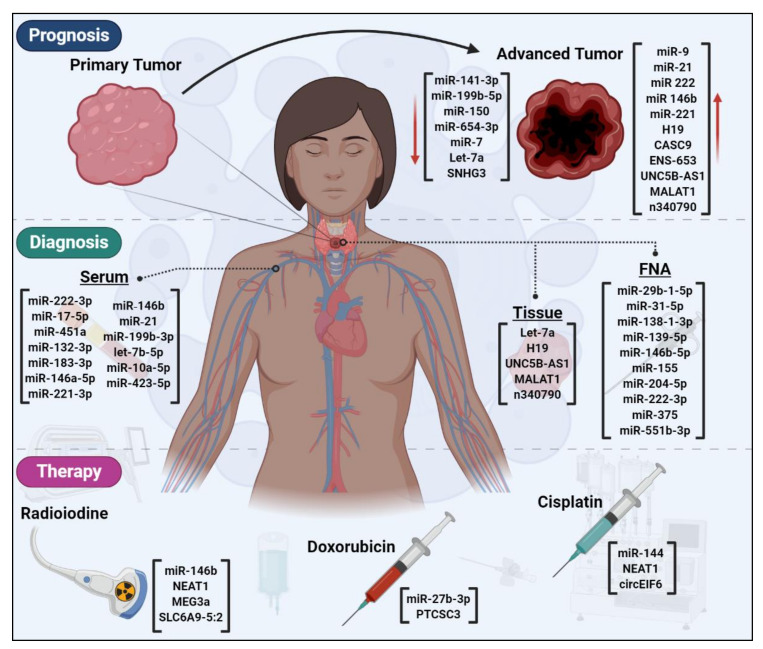
The diagnostic, prognostic and therapeutic significance of dysregulated ncRNAs in thyroid cancer. A variety of ncRNAs are dysregulated during primary to advanced tumor progression. These ncRNAs could have implications for the prognosis of thyroid cancer patients. The expression levels of ncRNAs could distinguish normal from tumor thyroid cells, therefore, acting as diagnostic biomarkers. Several ncRNAs may determine the response/resistance to the routine thyroid cancer treatment options of radioiodine and/or chemotherapy. FNA: fine needle aspiration.

Although the diagnostic values of miRNAs have been widely studied in the serum, the diagnostic significance of lncRNAs is dominantly studied in tissues. This may be because the stability of lncRNA levels is the lowest among several different RNA species [185]. A nonrandomized, retrospective study examining PTC patients and benign thyroid nodes revealed that lower H19 expression levels could distinguish PTC from benign with area under the curve (AUC) of the receiver operating characteristic (ROC) curve of 0.813 [127]. Liu et al. also studied the diagnostic significance of H19 together with let-7a in thyroid cancer patients showing that these ncRNAs could discriminate the thyroid cancer tumors against the healthy samples with AUC of 0.801 and 0.116 for H19 and let-7a, respectively [58]. The higher expression of UNC5B-AS1, MATAL1 and n340790 was shown to distinguish the tumor and normal samples with AUC values of 0.932, 0.632 and 0.845, respectively [119,123,124]. With the increasing identification of diagnostic circulating lncRNAs in different cancers [186,187,188,189,190], we expect to see the utilization of circulating lncRNAs to optimize diagnostic accuracy of thyroid nodules in the future. Collectively, ncRNAs could be clinically relevant biomarkers for thyroid cancer diagnosis (Figure 3).

Other than the ncRNAs utilized in the current commercial kit, other potential ncRNAs such as miR-146a-5p and H19 could be evaluated in clinical validation studies as diagnostic biomarkers. Although no circRNA has been reported to convey diagnostic significance in thyroid cancer, these ncRNAs have tremendous potential as diagnostic biomarkers due to the high stability of their circular structure and accumulation in exosomes. These characteristics result in the stable secretion of circRNAs in peripheral body fluids such as plasma and saliva, where they can be detected for early diagnosis of cancer [170,191,192,193].

## 7. ncRNAs as Prognostic Factors for Thyroid Cancer

ncRNAs have recently shown a massive capability as prognostic factors in human cancers [194,195]. For instance, the reduced expression of let-7 was shown to be associated with shortened postoperative survival of lung cancer patients [196]; and tumor suppressor candidate 7 (TUSC7) is a prognostic lncRNA that is inversely associated with aggressive stages and shorter survival of gastric cancer patients has been reported [197].

Thus far, BRAF^V600E^ and TERT [13]/p53 [198] mutations are the main molecular prognostic biomarkers [199] used along with clinicopathological factors such as age, extra-thyroid tumor spread, lymph node and distant metastases and increasing tumor size in thyroid cancer [200]. Nonetheless, nearly 30% of thyroid cancer patients may face over- or undertreatment in a condition based on BRAF status alone [200]. Moreover, the impact of BRAF status on the risk of recurrence in the very low-risk patients appears to be small [13]. This suggests that more molecular biomarkers are needed to determine the prognosis of thyroid cancer patients. In this section, we review the recent advances in prognostic ncRNAs in thyroid cancer, that are listed in Table 5.

The overexpression and oncogenic actions of miR-21 have been widely reported in thyroid cancer [54,204,205,206], suggesting that this miRNA could be a potential diagnostic factor for early detection of thyroid cancer. Beyond that, the association between miR-21 with the clinicopathological characteristics of thyroid cancer uncovered its capability to be used as a prognostic factor. The survival analysis by Zang et al. revealed that the higher expression of miR-21 can predict poor prognosis of PTC patients [54]. In another study, the multivariate survival analysis of patients for at least 120 months after surgery showed that miR-9 and miR-21 were significant independent prognostic factors for recurrence of PTC patients [201]. Zhang et al. studied miR-21 together with miR-221, miR-222 and miR-146b and depicted that all these miRNAs, except for miR-221, were highly expressed in poor-prognosis PTC patients group [174]. Comparing the expression level of miR-21, miR-222, miR-9, miR-10b, miR-146b, miR-31, miR-220 and miR-221 in recurrent vs. non-recurrent groups in another study showed that although all the miRNAs were dysregulated, only miR-221 overexpression was the independent prognostic factor of PTC recurrence [202]. The different outcomes among these studies could be due to the type of statistical tests used, e.g., multivariate vs. univariate Cox survival analysis. A deeper epidemiological analysis encompassing more dysregulated miRNAs, all the clinicopathological features and controlling for potential confounder parameters in a larger cohort size could better assess the utility of these miRNAs as prognostic markers.

miR-141-3p and miR-150 were separately demonstrated by different studies to be inversely associated with TNM stage and lymph node metastasis in PTC patients [61,64]. Similarly, the inverse association between miR-199b-5p or miR-7 and TNM stage was shown in PTC patients [66,69]. In another study, Geraldo et al. performed a prognosis study using the BRAF^V600E^-mutant PTC progression model in mice. The results interestingly showed that miR-654-3p levels underwent a significant decrease with long-term PTC progression in mice and negatively correlated with EMT. They further reported the down-regulation of miR-654-3p in PTC cell lines with the subsequent effect on increasing proliferation and migration. This suggests that not only this miRNA undergoes down-regulation in PTC development, but it also continues to be suppressed to progress cancer. Nonetheless, the epidemiological study is required to prove it as a prognostic factor in thyroid cancer [69].

With regard to the prognostic values of lncRNAs in thyroid cancer, several lncRNAs have been investigated. The decreased expression of small nucleolar RNA host gene 3 (SNHG3) was shown to correlate with the higher TNM stages and poorer prognosis of PTC patients [131]. On the contrary, CASC9, ENS-653, MALAT1 and UNC5B-AS1 associated positively with the advanced clinicopathological characteristics including large tumor size, advanced stage, or lymph node metastasis in PTC patients [114,116,123,124]. lncRNA H19 has been widely studied in different thyroid cancer cohorts that showed controversial prognostic results. In PTC, H19 was shown to be inversely associated with tumor size, pathological lateral node metastasis, extrathyroid extension, histological aggressive type and poorer disease-free survival [127]. The multivariate analysis of this retrospective, non-randomised study including 89 patients with benign thyroid nodes and 410 patients with PTC confirmed that H19 could be an independent risk factor for the extrathyroidal extension and lymph node metastasis [127]. Li et al. reported that higher H19 expression correlated with the poorer overall survival of PTC patients [118]. Similarly, Liu et al. studied thyroid cancer patients and showed that H19 positively correlated with higher TNM stages, lymph node metastasis and lower 5-year survival rate. Controlling for another ncRNA surveyed in this study, the higher H19 and lower let-7a along with tumor size, stage and lymph node metastasis were confirmed as the independent prognostic factors of thyroid cancer [58]. In FTC, H19 was revealed as the prognostic factor negatively associated with tumor size, distant metastasis and vascular invasion. However, the multivariate regression demonstrated that only age, primary tumor size ≥4 cm and vascular invasion were the significant prognostic factors of survival [126].

Taken together, the prognostic significance of ncRNAs has been shown in thyroid cancer (Figure 3). Notwithstanding, very little is known about the prognostic ncRNAs in more advanced thyroid cancers of MTC and ATC that show worse prognosis as compared to PTC and FTC. This indicates that the dysregulated ncRNAs could be studied epidemiologically in MTC/ATC cohorts.

## 8. ncRNAs Could Affect Thyroid Cancer Therapy

Upon thyroid cancer diagnosis, surgical excision of tumors is performed [13]. Patients with more advanced, differentiated thyroid cancers and a higher risk of recurrent or persistent disease undergo adjuvant RAI therapy. The molecular basis of this adjuvant therapy is the uptake of RAI via the plasma membrane sodium iodide symporter (NIS). NIS mediates the influx of RAI via transporting two Na^+^ ions and one I^−^ ion into the cytosol. RAI is then concentrated into the thyroid cells by iodine-metabolizing machinery. This eventually increases the efficiency of RAI therapy and improves the prognosis of thyroid cancer patients.

Notwithstanding, about 25–50% of locally advanced or metastatic thyroid cancers become refractory to RAI therapy. This leads to a poorer outcome with 5-year survival of <50% and 10-year survival of <10% [207,208]. RAI refractory response occurs through a complex de-differentiation process that leads to a diminished or a loss of NIS expression and/or correct localization. These prevent the cytoplasmic influx of RAI in thyroid cells, thereby causing adjuvant therapy resistance [209]. There are multiple mechanisms by which RAI resistance happens. Dysregulation of the MAPK signaling pathway is a well-studied mechanism that represses NIS protein expression. Thyroid cancer cells harboring BRAF^V600E^ mutations exhibit robust activation of MAPK signaling, associated with a de-differentiated state [209]. BRAF^V600E^-induced MAPK-independent repression of NIS has also been reported, where BRAF^V600E^ induces Transforming Growth Factor β (TGF-β) secretion. This resulted in the repression of NIS and elevated oncogenic properties in PTC cells [210]. The mechanistic role of PI_3_K and notch signaling pathways have also been demonstrated in RAI resistance [211]. Although the diversity of underlying mechanisms delineates the complexity and difficulty of the restoration of RAI sensitivity in thyroid cancer patients, different approaches have been implemented to overcome this clinical obstacle. Treatment of RAI-refractory thyroid cancer patients with retinoic acid [212], epigenetic transcriptional restoration of NIS expression via histone deacetylase inhibitors (HDACi) [213], or peroxisome proliferator-activated receptor (PPAR)-γ agonist [214] have shown sub-optimal re-differentiation outcomes for the patients.

Among various tested measures, ncRNAs have emerged as potential modulators of NIS restoration. Inhibition of miR-21, an upregulated miRNA in thyroid cancer, resulted in up-regulation of NIS expression, although the detailed mechanisms remain unknown [204]. In addition, miR-146b was reported to be highly up-regulated in dedifferentiated thyroid cancer cells, resulting in the repression of NIS via direct targeting of the NIS mRNA [215,216]. NIS expression can also be regulated by let7f-5p in PTC and FTC cells [217]. These findings highlight the potential clinical significance of inhibiting NIS-targeting miRNAs with respect to the re-differentiation and restoration of RAI sensitivity in RAI-refractory thyroid cancer patients. Additionally, transcriptome-wide approaches will be critical to identify the dysregulated lncRNAs and circRNAs in de-differentiated thyroid cancer cases, providing previously uncharacterized targets forpotential ncRNA-based strategies for the restoration of NIS expression. Given the known regulatory effects of miR-375, miR-497, CASC9 and XIST on the PI_3_K/Akt signaling pathway (Figure 2)—that is involved in NIS repression—in-depth studies could unravel their potential roles in the redifferentiation process.

Currently, patients with RAI-refractory differentiated thyroid cancer undergo systemic therapy, including targeted therapy and chemotherapy [12]. The chemotherapy drugs commonly used to treat thyroid cancer, in particular, the aggressive medullary and anaplastic thyroid malignancies are dacarbazine, vincristine, cyclophosphamide, doxorubicin, streptozocin, fluorouracil, paclitaxel, docetaxel and carboplatin [218,219,220,221,222]. However, chemotherapy is rarely used for thyroid cancer treatment, except for ATC patients. Doxorubicin was the only chemotherapy approved for the treatment of thyroid cancer patients. Nevertheless, it yielded a complete or partial response rate of <40% with limited durability [223]. Thus far, 2 multi-targeted tyrosine kinase inhibitors, sorafenib and lenvatinib, have been FDA-approved for the treatment of locally advanced or metastatic progressive RAI-refractory differentiated thyroid cancer. Both had been shown to improve progression-free survival but not overall survival [224,225,226]. For progressive metastatic MTC, 2 multi-targeted tyrosine kinase inhibitors, vandetanib and cabozantinib showed to improve progression-free survival and have been FDA-approved [227,228]. In metastatic BRAF^V600E^-mutant ATC, combined targeted therapy with dabrafenib and trametinib has been shown to improve progression-free survival and is FDA-approved for this indication [229]. Mutation-selective kinase inhibitor such as RET-inhibitor selpercatinib is FDA-approved for the treatment of metastatic RET-mutant MTC or RET-fusion mutant differentiated thyroid cancers with phase II clinical trial showing an overall response rate of 70%. Another mutation-selective kinase inhibitor that has been FDA-approved is the TRK inhibitor (larotrectinib or entrectinib) that can be used in metastatic thyroid cancers with NTRK-fusion mutation [230,231].

Serum thyroglobulin (Tg) is used as a tumor marker to monitor disease burden with treatment [232]. It detects recurrence in thyroid cancer with a sensitivity of 19–40% and specificity of 92–97% [233]. In addition, the presence of anti-thyroglobulin antibody in 25% of thyroid cancer patients affects the reliability of Tg assay [234,235]. Patients with poorly-differentiated thyroid cancers lose the ability to produce Tg, making the measurement of Tg an unreliable reflection of tumor burden [232]. Given the low survival rate of aggressive thyroid cancer patients and the rather low sensitivity of Tg for detecting thyroid cancer recurrence leave room for the development of molecular tools that are more sensitive, and hopefully equally specific, than Tg.

The other underlying factor playing role in cancer treatment failure could be drug resistance, leading to elevated cancer relapse and mortality in patients [236,237]. ncRNAs are increasingly studied to unravel the complex mechanism of drug resistance development [238,239]. In this section, we discuss the recent advances contributing to the understanding of how ncRNAs contribute to drug sensitivity and/or resistance in thyroid cancer (Table 6).

Icariin, a chemical flavonoid compound isolated from different species of the genus *Epimedium* plant, has recently emerged as an anti-cancer substance [244], e.g., in ovarian cancer by targeting miR-21 [245] or in colorectal cancer through enhancing the NFκB suppression-mediated radiosensitivity [246]. In thyroid cancer, Fang et al. demonstrated that icariin inhibited cell growth, invasion and migration, while promoting apoptosis [56]. Mechanistically, icariin was shown to target miR-625-3p leading to inactivation of the PI_3_K/Akt and mitogen-activated protein kinase kinase (MEK)/mitogen-activated protein kinase 1 (ERK) signaling pathways [56]. Therefore, targeting miR-625-3p expression that is elevated in thyroid cancer could enhance the therapeutic sensitivity of tumor cells to icariin. In another study, Xu et al. reported that miR-27b-3p expression level was increased in doxorubicin-resistant ATC cells through targeting and suppressing peroxisome proliferator-activated receptor gamma (PPARγ) gene [240]. Their findings indicated that targeted inhibition of miR-27b-3p might be a potential therapeutic approach in doxorubicin-resistant ATC cells. Another study on progressive ATC cells depicted that down-regulation of miR-144 led to the cisplatin resistance. Transfection with miR-144 mimics improved the sensitivity of ATC cells to cisplatin and inhibited tumor growth by suppressing (transforming growth factor alpha) TGF-α both in vitro and in vivo [62]. Collectively, the dysregulation of miRNAs could determine the sensitivity or resistance to the anti-tumor compounds in thyroid cancer (Figure 3). Together with drug screening, the miRNA microarray array clinical studies could provide more insights into the functional miRNAs involved in drug response.

To date, many lncRNAs have been shown in human cancer to play role as therapeutic determinants [247,248,249]. In thyroid cancer, lncRNAs have recently emerged as important factors involved in the sensitivity of patients to different treatments. LncRNA NEAT1 has been shown to have therapeutic implications in different thyroid cancer types. In ATC, in vitro and in vivo overexpression of NEAT1 was demonstrated to elevate cisplatin resistance via sponging miR-9-5p, which resulted in rescuing perm associated antigen 9 (SPAG9) [67]. In addition, higher expression of NEAT1 was detected in radioactivity iodine-resistant PTC tissues and cell lines, and this was associated with miR-101-3p inhibition, Fibronectin 1 overexpression, and PI_3_K/Akt pathway activation [242]. These highlight the importance of targeted inhibition of NEAT1 to overcome the chemotherapy and radioactivity iodine-resistance of thyroid cancer patients.

In addition to NEAT1, solute carrier family 6 member 9–5:2 (SLC6A9–5:2) and maternally expressed 3 (MEG3a) were discovered as therapeutic lncRNAs in determining the response to ^131^I therapy. MEG3a, an important lncRNA in different human diseases [250,251,252], was shown to be down-regulated in radioactivity iodine-resistant PTC and FTC tissues and cell lines. Mechanistically, overexpression of MEG3a resulted in sponging miR-182 that subsequently suppressed ^131^I-resistant cell viability and induced DNA damage [241]. Similarly, SLC6A9-5:2 down-regulation was reported in radioactivity iodine-resistant PTC tissues and cell lines. However, overexpression of SLC6A9-5:2 revoked the ^131^I-resistant sensitivity via up-regulating PARP-1 protein with an unknown mechanism [243]. lncRNA PTCSC3 was observed to be down-regulated in ATC tissues and cell lines leading to increased STAT3 and INO80 expression. This axis consequently conferred resistance to doxorubicin [130]. This indicates that overexpressing PTCSC3 could overcome the resistance to doxorubicin in ATC patients.

The physiological roles of circRNAs are not restricted to their contribution to cancer development. circRNAs, could also determine the drug response and thereby promote drug resistance in human cancers [253,254,255,256,257]. In thyroid cancer, circular eukaryotic translation initiation factor 6 (circEIF6) has been shown to inhibit the response to cisplatin. In the presence of this chemotherapeutic drug, circEIF6 was shown to inhibit the apoptosis via sponging miR-144-3p and the corresponding up-regulation of TGF-α, thereby enhancing the resistance to cisplatin in both PTC and ATC thyroid cancer cells [142].

Collectively, ncRNAs play role as therapeutic factors in thyroid cancer (Figure 3). Altering miR-146b, NEAT1, MEG3a, SLC6A9-5:2 may synergistically improve the resistance to radioactive iodine, while targeted inhibition of miR-27b-3p and PTCSC3 may overcome the resistance to doxorubicin. To heighten the sensitivity to cisplatin, miR-144, NEAT1 and circEIF6 could be studied together in thyroid cancer. Future studies could evaluate the functional role of ncRNAs in determining the response to targeted kinase therapy.

## 9. Conclusions and Future Perspectives

Over the years, remarkable progress has been achieved in mapping the genetic basis of thyroid cancer and developing more efficient molecular tests for its early detection. Notwithstanding, the overall survival of MTC, ATC, PDTC and metastatic differentiated thyroid cancer patients have not improved satisfactorily, reflecting the need for deciphering pristine molecular determinants that could guide early diagnosis and personalized treatment- utilization of ncRNAs is a promising strategy.

In this review, we discussed the current knowledge of three main subtypes of ncRNAs, including miRNAs, lncRNAs and circRNAs in different histopathological subtypes of thyroid cancer. Dysregulation of ncRNAs play an important role in thyroid cancer pathogenesis. This information could be utilized in the diagnostic, prognostic and therapeutic aspects of thyroid cancer clinical care. A number of clinical trials are ongoing to investigate the potential diagnostic and therapeutic impact of ncRNA molecules in thyroid cancer (Table 7).

Owing to the tumor-suppressive or oncogenic function, dysregulated ncRNAs could promote tumorigenesis via regulating various physiological and cellular activities leading to proliferation/cell growth or inhibition of cell death. However, little is known about the detailed regulatory mechanisms by which the ncRNAs, especially circRNAs, are dysregulated in thyroid cancer with the corresponding downstream tumorigenic and drug-resistance cascades. Understanding the mode of action by which different ncRNAs, individually or in a network, impose their oncogenic effects could aid in the development of new therapeutic approaches to harness the progression of malignant cells. This could be primarily achieved by rational in vitro RNA-based drug design to target the up-regulated ncRNAs using antagomirs and antisense oligos (ASOs), or by expressing the key down-regulated ncRNAs using agomirs and expression vectors. However, selecting the key target ncRNAs from a large number of candidate ncRNAs remains a big challenge.

The other challenge would be the instability and high immunogenicity of the RNA therapeutics, necessitating chemical modifications of the RNA molecules. One example is using the inverted thymidine residues at the 3′ end of the RNA to protect it against exonucleases, thereby improving stability [258]. In addition, conjugating the RNA with an active targeting moiety, such as an antibody, has been shown to reduce immunogenicity [259]. Although significant progress has been made, the delivery of RNA therapeutics remains a major challenge. Negatively charged phosphate backbones and large molecular weight of RNA molecules hamper RNA uptake through difficulties in passing through the cell membrane, micropinocytosis, endosomal escape and kidney clearance [258,260,261]. In addition to reducing immunogenicity, the conjugation of RNAs with targeting moieties including antibodies, aptamers, lipic nanoparticles and polymers has led to magnificent advances in the delivery efficiency of RNA therapeutics [262]. Attachment of a monoclonal antibody (TCM-9), a specific antibody for human thyroid cancer [263], could be a strategy to improve the targeted delivery of RNA therapeutics to thyroid carcinoma cells.

Combination of ncRNAs-based therapeutic interventions with conventional systemic therapy could emerge as an impactful way to conquer drug resistance in advanced thyroid cancer. Such in-depth studies may prove the way toward pre-clinical and clinical investigations that eventually could provide more impactful therapies. Beyond the understanding of cancer pathogenesis and drug resistance, the alteration in circulating or tissue ncRNAs expression could facilitate the diagnosis of different thyroid malignancies with improved sensitivity and specificity, and a minimized need for diagnostic thyroid surgeries. Taken together, we expect the application of ncRNAs as diagnostic/prognostic biomarkers and therapeutic targets to emerge within the few next years in thyroid cancer.

## Figures and Tables

**Table 1:** Dysregulated miRNAs in thyroid cancer.

miRNA	Alteration	Mechanism	Thyroid Cancer Type	Sample	Ref.
miR-146a/b-5p	↑	↓ RAR-β	PTC	Cell line and tissue	[52]
miR-155		↓ SOCS1	ATC		[53]
miR-21		↓ VHL → ↑ N-cadherin and vimentin	PTC		[54]
miR-574-5p		↓ SCAI → ↑ β-catenin			[55]
miR-625-3p		↑ Bcl-2, ↓ Bax, and cleaved caspase 3/9			[56]
miR-96		↓ FOXO1 → ↓ Bim		Cell line	[57]
Let-7a	↓	Not known	Not known	Tissue	[58]
miR-125a-5p		↑ CD147 → ↓ glucose metabolism	PTC, FTC, MTC and ATC	Cell line and tissue	[59]
miR-1266		↑ FGFR2	PTC		[60]
miR-141-3p		↑ YY1			[61]
miR-144		↑ TGF-α	ATC		[62]
miR-148a		↑ INO80		ATC cancer stem cells	[63]
miR-150		↑ROCK1	PTC	Cell line and tissue	[64]
miR-195		↑ CCND1 and FGF2 → ↑ β-catenin, c-Myc, cyclin D1 and MMP-13			[65]
miR-199b-5p		↑ STON2 → ↑ N-cadherin and fibronectin			[66]
miR-375		↑ ERBB2			[57]
miR-497		↑ AKT3			[67]
miR-524-5p		↑ FOXE1 and ITGA3			[68]
miR-7		↑ PAK1			[69]
miR-873-5p		↑ CXCL16 → ↑ p-p65 and p-Rel-B, MMP1, MMP9 and MMP13			[70]
miR-9-5p		↑ BRAF			[67]

PTC: Papillary Thyroid Cancer; FTC: Follicular Thyroid Cancer; ATC: Anaplastic Thyroid Cancer; MTC: Medullar Thyroid Cancer; FOXN3: Forkhead Box N3; QKI5-7: Quaking protein 5-7; SCAI: Suppressor of Cancer Cell Invasion; CXCL16: C-X-C Motif Chemokine Ligand 16; SOCS1: Suppressor Protein of Cytokine Signaling 1; MMP1: Matrix Metallopeptidase 1; VHL: Von Hippel-Lindau Tumor Suppressor; RAR-β: Retinoic Acid Receptor Beta; FOXE1: Forkhead Box E1; ITGA3: Integrin Subunit Alpha 3; HK2: Hexokinase 2; YY1: Yin Yang 1; STON2: Stonin 2; FGFR2: Fibroblast Growth Factor Receptor 2; TGF-α: Transforming Growth Factor Alpha; AKT3: RAC-γ serine/threonine-protein kinase; CCND1: Cyclin D1; FGF2: Fibroblast Growth Factor; ROCK1: Rho-associated Protein Kinase 1; PAK1: p21 Activated Kinase-1; ERBB2: Erb-B2 Receptor Tyrosine Kinase 2; FOXO1: Forkhead Box O1.

**Table 2:** Dysregulated lncRNAs in thyroid cancer.

LncRNA	Alteration	Mechanism	Thyroid Cancer Type	Sample	Ref.
CASC9	↑	↓ miR-488-3p → ↑ ADAM9 → ↑ EGFR/PI_3_K/Akt pathway activation.	PTC	Cell line and tissue	[114]
DLX6-AS1		Negatively correlated with UPF1	Not known		[115]
ENST00000539653.1 (ENS-653)		Not known	PTC	Tissue	[116]
H19		Not known	ATC		[117]
		Not known	Not known		[58]
		↓ miR-3126-5p → ↑ ER-β	PTCSCs and PTC tissue		[118]
HCP5		↓ miR-22-3p, miR-186-5p and miR-216a-5p → ↑ ST6GAL2	FTC	Cell line and tissue	[119]
LINC00152		↓ miR-497 → ↑ BDNF	PTC		[120]
LINC00514		↓ miR-204-3p → ↑ CDC23			[121]
LINC00941		↓ CDH6			[122]
MALAT1		No mechanism			[123]
n340790		↓ miR-1254	Not known		[119]
NEAT1		↓ miR-9-5p ↑ SPAG9	ATC		[67]
UNC5B-AS1		Not known	PTC	Tissue	[124]
XIST		↓ miR-34a → ↑ MET → PI_3_K/Akt activation	Not known	Cell line and tissue	[125]
H19	↓	Not known	FTC	Tissue	[126]
H19		Not known	PTC		[127]
LINC003121		↑ PI_3_K and p-Akt	Not known	Cell line and tissue	[128]
PAR5		↑ EZH2 → ↓ E-cadherin	ATC		[129]
PTCSC3		↑ STAT3 → ↑ INO80			[130]
		↑ miR-574-5p → ↓ SCAI → ↑ β-catenin → ↑ Wnt pathway activation	PTC		[55]
SNHG3		↑ PI_3_K/Akt/mTOR pathway			[131]

PTC: Papillary Thyroid Cancer; FTC: Follicular Thyroid Cancer; ATC: Anaplastic Thyroid Cancer; PTCSCs: Papillary Thyroid Cancer Stem Cells; SNHG3: Small Nucleolar RNA Host Gene 3; PAR5: Prader Willi/Angelman Region RNA5; CASC9: Cancer Susceptibility 9; DLX6-AS1: Distal-Less Homeobox 6-Antisense 1; NEAT1: Nuclear Paraspeckle Assembly Transcript 1; UNC5B-AS1: Unc-5 Netrin Receptor B-Antisense 1; XIST: X-inactive specific transcript; MALAT1: Metastasis Associated Lung Adenocarcinoma Transcript 1; HCP5: HLA complex P5; PTCSC3: Thyroid Carcinoma Susceptibility Candidate 3; PI_3_K: Phosphatidylinositol-4,5-Bisphosphate 3-Kinase; mTOR: Mechanistic Target of Rapamycin Kinase; EZH2: Enhancer of Zeste Homolog 2; ADAM9: ADAM Metallopeptidase Domain 9; EGFR: Epidermal Growth Factor Receptor; UPF1: UPF1 RNA Helicase and ATPase; ER-β: Estrogen Receptor Beta; SPAG9: Perm-Associated Antigen 9; BDNF: Brain-Derived Neurotrophic Factor; CDH6: Cadherin 6; STAT3: Signal Transducer And Activator of Transcription 3; ST6GAL2: alpha-2, 6-sialyltransferase 2; SCAI: Suppressor of Cancer Cell Invasion.

**Table 3:** Dysregulated circRNAs in thyroid cancer.

circRNA	Alteration	Mechanism	Sample	Thyroid Cancer Type	Ref.
circ_0008274	↑	↓ AMPK/mTOR signaling pathway	Cell line and tissue	PTC	[141]
circEIF6	↑	↓ miR-144-3p → ↑ TGF-α	Cell line and tissue	PTC & ATC	[142]
circFOXM1	↑	↓ miR-1179 → ↑ HMGB1	Cell line and tissue	PTC	[143]

PTC: Papillary Thyroid Cancer; ATC: Anaplastic Thyroid Cancer; circFOXM1: Circular Forkhead Box Protein M1; circEIF6: circular Eukaryotic Translation Initiation Factor 6; HMGB1: High Mobility Group Box Protein 1; AMPK: 5′ AMP-activated Protein Kinase; mTOR: Mammalian Target of Rapamycin (mTOR); TGF-α: Transforming Growth Factor Alpha.

**Table 4:** Diagnostic ncRNAs in thyroid cancer.

ncRNA	ncRNA Type	Source	Finding	Thyroid Samples	Ref.
miR-138-1-3p miR-139-5p miR-146b-5p miR-155miR-204-5p miR-222-3pmiR-29b-1-5p miR-31-5p miR-375 miR-551b-3p	miRNA	FNA	miRNA testing, recently commercialized as ThyraMIR, identified 64% of malignant cases and 98% of benign cases correctly.	Not known	[170]
miR-146b			Higher expression in PTC FNA samples	PTC	[171]
miR-132-3p miR-146a-5pmiR-17-5p miR-183-3p miR-222-3p miR-451a		Serum	miR-222-3p and miR-17-5p can accurately discriminate MTC from the benign nodule and healthy control groups	PTC, MTC, benign nodules and controls	[172]
miR-146a-5p miR-221-3p miR-222-3p			High pre-surgical expressionLow post-surgical expression	PTC	[173]
miR-146b miR-21 miR-221 miR-222			Higher expression in PTM serum samples		[174]
miR-146a-5p miR-199b-3p			Lower expression in PTC serum as compared to benign serum samples		[175]
let-7b-5pmiR-10a-5p			Higher expression in PTC serum as compared to benign serum samples		
miR-423-5p			Higher expression in PTC serum samples		[176]
let-7a		Tissue	Lower expression in thyroid tumor samples	Not known	[58]
H19	lncRNA		Higher expression in thyroid tumor samples		
H19			Lower expression in thyroid tumor samples as compared to benign samples	PTC	[127]
MALAT1			Higher expression in thyroid tumor samples		[123]
n340790				Not known	[119]
UNC5B-AS1				PTC	[124]

PTC: Papillary Thyroid Cancer; MTC: Medullar Thyroid Cancer; UNC5B-AS1: Unc-5 Netrin Receptor B-Antisense RNA 1; MALAT1: Metastasis Associated Lung Adenocarcinoma Transcript 1; FNA: Fine Needle Aspiration.

**Table 5:** Prognostic ncRNAs in thyroid cancer.

ncRNA	ncRNA Type	Prognostic Significance	Thyroid Cancer Type	Ref.
Let-7a	miRNA	Negative correlation with higher TNM stages lymph node metastasis and lower 5-year survival	Not known	[58]
miR-141-3p		Negative association with TNM stage and lymph node metastasis	PTC	[61]
miR-146b miR-21 miR-222		Poor prognosis		[174]
miR-150		Negative association with TNM stage and lymph node metastasis		[64]
miR-199b-5p		Negative association with stage		[66]
miR-21		Poor prognosis		[54]
miR-21 miR-9		Independent prognostic factors of PTC recurrence		[201]
miR-221		Independent prognostic factors of PTC recurrence		[202]
miR-654-3p		Down-regulation upon a long-term PTC progression in BRAF^V600E^-transgenic mice		[203]
miR-7		Negative association with stage		[69]
CASC9	lncRNA	Positive association with large tumor size, advanced stage, or lymph node metastasis.		[114]
ENST00000539653.1 (ENS-653)		Positive association with larger tumor size, more advanced clinical stage and poorer disease-free survival		[116]
H19		Positive correlation with higher TNM stages lymph node metastasis and lower 5-year survival	Not known	[58]
		Positive correlation with poor overall survival	PTCSCs and PTC tissue	[118]
		Negative correlation with extrathyroid extension, tumor size, histological aggressive type, pathological lateral node metastasis and poorer disease-free survivalIndependent risk factor for extrathyroidal extension and lymph node metastasis.	PTC	[127]
		Negative association with tumor size, distant metastasis and vascular invasion	FTC	[126]
MALAT1		Positive correlation with tumor size and lymph node metastases	PTC	[123]
n340790		Positive correlation with primary clinicopathological characteristics (good prognostic factor)	Not known	[119]
SNHG3		Negative association with stage and poor prognosis	PTC	[131]
UNC5B-AS1		Positive correlation with lymph node metastasis, tumor size and histological type		[124]

PTC: Papillary Thyroid Cancer; FTC: Follicular Thyroid Cancer; ATC: Anaplastic Thyroid Cancer; PTCSCs: Papillary Thyroid Cancer Stem Cells; SNHG3: Small Nucleolar RNA Host Gene 3; CASC9: Cancer Susceptibility 9; PTCSC3: Thyroid Carcinoma Susceptibility Candidate 3; UNC5B-AS1: Unc-5 Netrin Receptor B-Antisense RNA 1; MALAT1: Metastasis Associated Lung Adenocarcinoma Transcript 1.

**Table 6:** Therapeutic ncRNAs in thyroid cancer.

ncRNA	ncRNA Type	Therapeutic Significance	Thyroid Cancer Type	Ref.
miR-144	miRNA	↑ Sensitivity to cisplatin	ATC	[62]
miR-146b		↓ Radioiodine uptake	FTC	[215]
miR-27b-3p		↑ Resistance to doxorubicin	ATC	[240]
miR-625-3p		Target of icariin anti-tumor substance	PTC	[56]
MEG3a	lncRNA	↑ Resistance to radioactive iodine	FTC and PTC	[241]
NEAT1		↑ Resistance to cisplatin	ATC	[67]
NEAT1		↑ Resistance to radioactive iodine	PTC	[242]
PTCSC3		↑ Resistance to doxorubicin	ATC	[130]
SLC6A9-5:2		↑ Resistance to radioactive iodine	PTC	[243]
circEIF6	circRNA	↑ Resistance to cisplatin	PTC and ATC	[142]

FTC: Follicular Thyroid Cancer; PTC: Papillary Thyroid Cancer; ATC: Anaplastic Thyroid Cancer; NEAT1: Nuclear Paraspeckle Assembly Transcript 1; MEG3a: Maternally Expressed Gene 3; PTCSC3: Papillary Thyroid Carcinoma Susceptibility Candidate 3; SLC6A9-5:2: Solute Carrier Family 6 Member 9-5:2; circEIF6: Circular Eukaryotic Translation Initiation Factor 6.

**Table 7:** List of ongoing clinical trials indexed in ClinicalTrials.gov (https://clinicaltrials.gov/ct2/home) portal with diagnostic or prognostic relevance in thyroid cancer.

Identifier	ncRNA Type	Type of Sample	Study Type	Observational Model	Clinical Significance	Status
NCT03469544	HOTAIR	Peripheral blood samples	Observational	Case-Control	Diagnostic biomarker	Not yet recruiting
NCT01964508	miRNAs	FNA samples	Observational	Cohort	Diagnostic biomarker	Not yet recruiting
NCT04594720	lncRNAs	Peripheral blood samples	Observational	Case-Control	Diagnostic biomarker	Recruiting completed
NCT01240590	miRNAs	ATC tumor samples	Interventional	Parallel Assignment	Therapeutic biomarker for Crolibulin and cisplatin	Recruiting completed
NCT04285476	miRNAs	Thyroid carcinoma	Interventional	Single Group Assignment	Diagnostic biomarker	Not yet recruiting
NCT00689065	siRNA	Variety of solid tumors including Thyroid carcinoma	Interventional	Single Group Assignment	RNA-based therapy (CALAA-01)	Recruitment terminated

FNA: fine needle aspiration.

## Abbreviations

ADAM9	ADAM Metallopeptidase Domain 9
AKT3	RAC-γ serine/threonine-protein kinase
AMPK	5′ AMP-activated Protein Kinase
ATC	Anaplastic Thyroid Cancer
BDNF	Brain-Derived Neurotrophic Factor
CASC9	Cancer Susceptibility 9
CCND1	Cyclin D1
CDH6	Cadherin 6
circEIF6	Circular Eukaryotic Translation Initiation Factor 6
circFOXM1	Circular Forkhead Box Protein M1
circRNA	Circular RNA
CXCL16	C-X-C Motif Chemokine Ligand 16
DLX6-AS1	Distal-Less Homeobox 6-Antisense 1
EGFR	Epidermal Growth Factor Receptor
ERBB2	Erb-B2 Receptor Tyrosine Kinase 2
ER-β	Estrogen Receptor Beta
EZH2	Enhancer of Zeste Homolog 2
FGF2	Fibroblast Growth Factor
FGFR2	Fibroblast Growth Factor Receptor 2
FNA	Fine Needle Aspiration
FOXE1	Forkhead Box E1
FOXN3	Forkhead Box N3
FOXO1	Forkhead Box O1
FTC	Follicular Thyroid Carcinoma
HCP5	HLA complex P5
HK2	Hexokinase 2
HMGB1	High Mobility Group Box Protein 1
ITGA3	Integrin Subunit Alpha 3
lncRNA	Long Non-coding RNA
MALAT1	Metastasis Associated Lung Adenocarcinoma Transcript 1
MEG3a	Maternally Expressed Gene 3
miRNA	microRNA
MMP1	Matrix Metallopeptidase 1
MTC	Medullary Thyroid Carcinoma
mTOR	Mechanistic Target of Rapamycin Kinase
ncRNA	Non-coding RNA
NEAT1	Nuclear Paraspeckle Assembly Transcript 1
PAK1	p21 Activated Kinase-1
PAR5	Prader Willi/Angelman Region RNA5
PDTC	Poorly differentiated thyroid carcinoma
PI_3_K	Phosphatidylinositol-4,5-Bisphosphate 3-Kinase
piRNA	PIWI-interacting RNA:
PTC	Papillary Thyroid Cancer
PTCSC3	Thyroid Carcinoma Susceptibility Candidate 3
PTCSCs	Papillary Thyroid Cancer Stem Cells
QKI5-7	Quaking protein 5-7
RAR-β	Retinoic Acid Receptor Beta
ROCK1	Rho-associated Protein Kinase 1
SCAI	Suppressor of Cancer Cell Invasion
SEER	Surveillance, Epidemiology and End Results
SLC6A9-5:2	Solute Carrier Family 6 Member 9-5:2
SNHG3	Small Nucleolar RNA Host Gene 3
snoRNA	Small nuclear RNA
SOCS1	Suppressor Protein of Cytokine Signaling 1
SPAG9	Perm Associated Antigen 9
ST6GAL2	alpha-2, 6-sialyltransferase 2
STAT3	Signal Transducer And Activator of Transcription 3
STON2	Stonin 2
TGF-α	Transforming Growth Factor Alpha
UNC5B-AS1	Unc-5 Netrin Receptor B-Antisense RNA 1
UPF1	UPF1 RNA Helicase and ATPase
VHL	Von Hippel-Lindau Tumor Suppressor
XIST	X-inactive specific transcript
YY1	Yin Yang 1
